# BCM-7: Opioid-like Peptide with Potential Role in Disease Mechanisms

**DOI:** 10.3390/molecules29092161

**Published:** 2024-05-06

**Authors:** Ecem Bolat, Furkan Eker, Selin Yılmaz, Sercan Karav, Emel Oz, Charles Brennan, Charalampos Proestos, Maomao Zeng, Fatih Oz

**Affiliations:** 1 Department of Molecular Biology and Genetics, Çanakkale Onsekiz Mart University, Çanakkale 17100, Türkiye; ecemmbolatt@gmail.com (E.B.); furkanekeerrr@gmail.com (F.E.); selinyilmaz153@gmail.com (S.Y.); 2 Department of Food Engineering, Faculty of Agriculture, Atatürk University, Erzurum 25030, Türkiye; emel.oz@atauni.edu.tr (E.O.); fatihoz@atauni.edu.tr (F.O.); 3 School of Science, RMIT University, Melbourne, VIC 3001, Australia; charles.brennan@rmit.edu.au; 4 Laboratory of Food Chemistry, Department of Chemistry, School of Sciences, National and Kapodistrian University of Athens Zografou, 157 84 Athens, Greece; harpro@chem.uoa.gr; 5 State Key Laboratory of Food Science and Resources, Jiangnan University, Wuxi 214122, China; mmzeng@jiangnan.edu.cn

**Keywords:** β-casomorphin-7, A1 milk, A2 milk, opioid peptide, health effect

## Abstract

Bovine milk is an essential supplement due to its rich energy- and nutrient-rich qualities. Caseins constitute the vast majority of the proteins in milk. Among these, β-casein comprises around 37% of all caseins, and it is an important type of casein with several different variants. The A1 and A2 variants of β-casein are the most researched genotypes due to the changes in their composition. It is accepted that the A2 variant is ancestral, while a point mutation in the 67th amino acid created the A1 variant. The digestion derived of both A1 and A2 milk is BCM-7. Digestion of A2 milk in the human intestine also forms BCM-9 peptide molecule. The opioid-like characteristics of BCM-7 are highlighted for their potential triggering effect on several diseases. Most research has been focused on gastrointestinal-related diseases; however other metabolic and nervous system-based diseases are also potentially triggered. By manipulating the mechanisms of these diseases, BCM-7 can induce certain situations, such as conformational changes, reduction in protein activity, and the creation of undesired activity in the biological system. Furthermore, the genotype of casein can also play a role in bone health, such as altering fracture rates, and calcium contents can change the characteristics of dietary products. The context between opioid molecules and BCM-7 points to a potential triggering mechanism for the central nervous system and other metabolic diseases discussed.

## 1. Introduction

### 1.1. Cow Milk

Bovine milk consists of various combinations of protein, lactose, fat, water, and trace amounts of other components [1,2]. It also contains a substantial amount of vitamins, and minerals such as vitamin B12, vitamin A, iron, calcium, potassium, and phosphorus [3]. These microcomponents are essential for the body [1], serving as a comprehensive supplement for children during their developmental period and have a positive effect on adults as well [4]. In broader terms, milk supports the intestine and immune system as a prebiotic substance, thus reducing the risk of diabetes and cardiovascular diseases [5,6]. For these reasons, milk formula is often introduced as the first food for infants in the absence of breastfeeding or in case of malnutrition [1]. While caseins constitute 80% of milk proteins, whey proteins constitute 20%. Cow’s milk contains four different casein types, and the second most abundant protein, with 37%, is β-caseins [7]. β-casein possesses balanced amino acid nutritional ratio and is useful in maintaining a healthy cycle within the body. β-casein occurs in 13 different variants, with the most commonly known variants being A1 and A2 [8,9,10]. Milk can be classified according to its casein type, primarily due to its various physicochemical properties.

### 1.2. A1 & A2 Milk

**Figure 1:**
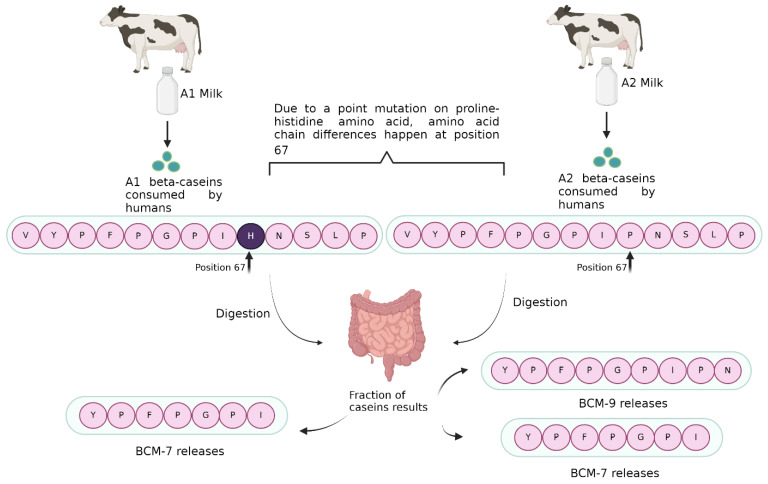
Formation of BCM-7 and BCM-9 cow’s milk peptides.

The protein content of bovine milk consists of two main types: 80% caseins and 20% whey proteins [11]. Caseins comprise 45% of bovine milk proteins and can be categorized into four types: as1, as2, β, and k [12]. Among them, β-casein holds numerous important functions [13]. A1 and A2 variants consist of two alleles for the β-casein gene, like A1/A1, A2/A2, or A1/A2 combinations, which are possible because of a natural mutation about 8,000 years ago. These variants are genetically transmitted from parents to offspring. Different studies have determined A1 and A2 allele [14,15]. Furthermore, the A1 variant is associated with a genetic selection [16]. In a different study, a specific phenotype of β-casein was determined from 24 different crossbred (A1/A2) Jersey breeds. As a result of this study, which is a proteomic approach, proline and histidine residues were highlighted in the proteome database of A1 and A2 β-casein [10]. Originally, the β-casein protein comprised 209 amino acid chains in the A2 variant. However, the A1 variant emerged due to a natural mutation occurring in the A2 variant’s amino acid chain at the 67th position (Figure 1) [17]. This mutation, a point mutation, changes the original proline (Pro67) at position 67 to the amino acid histidine (His67). This alteration in a single amino acid disturbs the three-amino-acid codon, changing CCT to CAT. This shift affects various aspects, including enzyme target sites and the ratios of lactose and fatty acids between the variants [13].

Due to the distinction in the nucleotide code between these β-casein variants, the two variants differ in terms of chemical and biological effects. These effects become noticeable over the long term when a specific type of milk is consumed consistently due to the digestion of the A1 and A2 variants. Upon digestion, two peptides with different structures are released. Digestion from A1 and A1/A2 variant milk yields β-casomorphin-7 (BCM-7), whereas digestion of A2 variant milk releases β-casomorphin-9 (BCM-9) and a low amount of β-casomorphin-7 [8,18]. However, the one-terminal structure of the BCM-7 peptides is not the same as that of A1 milk-derived BCM-7. This may affect the BCM-7’s capabilities like absorption, transport, and binding to opioid receptors [19].

### 1.3. β-Casomorphin-7 and It’s Opioid Receptor Agonist Character

Casemorphins were first discovered in the late 1970s for their opioid-like properties [20,21]. The β-casomorphins (BCMs) are exogenous peptides released during digestion that can function as opioid-like molecules, acting as opioid receptor agonists [22]. These peptides can be any opioid protein structures formed during the digestion of casein, and the casomorphins relevant to human health are those formed from β-caseins. β-caseins are present in the milk of all mammals, including bovine milk. There are nine different BCMs in bovine milk: BCM-3 (YPF), BCM-4 (YPFP), BCM-5 (YPFPG), BCM-6 (YPFPGP), BCM-7 (YPFPGPI), BCM-8 (YPFPGPIP/H), BCM-9 (YPFPGPIPN), BCM-10 (YPFPGPIPNS), BCM-11 (YPFPGPIPNSL) [23,24,25]. Two of the prominent β-casomorphins (BCMs) are BCM-7 and the longer BCM-9 [26].

A2 β-casein is less likely to be cleave and form the BCM-9 protein, which is differs from BCM-7 primarily due to differences in amino acid chain position, and BCM-9 with a proline at position 67 is cleavage-resistant [27]. Because A2 milk polymorphism is unfavorable against enzymatic cleavage, when compred to A1, the product of the digestion will be favorably BCM-7 protein. In other words, when the consumption of milk is high in the regions that have a high frequency of A1 polymorphism cows, the accumulation of β-casomorphin-7 will be more likely [28]. On the other hand, the consumption of BCM-9 (which indicates the high frequency of A2 polypmorphism) has been related to favorable health effects in humans, such as decreased gastrointestinal symptoms [29,30]. Digestion of the A1 β-casein variant, which can be one of the causes of common lactose intolerance or milk allergy [31], has also been shown to have the potential to trigger the onset of multiple diseases (Table 1). 

“Opioid Food Peptides”, a book that was published in 2020, briefly discussed the opioid activity of casomorphins [32]. As discussed, BCM shows affinity towards µ-opioid receptors, independent of the source (in the case of humans or bovines). Still, the results of the comparative studies indicate that bovine BCMs show higher potency than human BCMs. It was indicated that human BCMs show up to 30 times less potency than bovine BCMs. 

Food-derived peptides or proteins can affect physiological functions (such as antioxidant, antimicrobial, immunomodulator, and modulation of intestinal secretion) [33,34]. Some of these proteins may be categorized as opioid peptides due to their high affinity for opioid receptors and opiod-like effects upon receptor interaction [35]. Among these, BCM-7 is a pro-inflammatory exogenous (food protein) opioid peptide. β-casomorphins bind to an µ-opioid receptor in mainly the gastrointestinal tract and central nervous system [8]. Opioid receptors have different types, which are distributed in different parts of the brain. Most bovine BCMs, including BCM-7 and BCM-5, are selective to µ-opioid receptors and show agonist interaction. On the other hand, human BCMs, including BCM-7 and BCM-5, are also selective to *µ*-opioids. However, their selectivity is not limited to µ-opioid like-bovine BCMs. They are also selective for delta (second) and kappa (third) receptors, which are lower compared to µ-opioid receptor types [36]. When the activity of these BCMs was observed in guina-pig ileum and mouse vas deferens, bovine BCM-7 shows the highest opioid activity among other types of bovine and human BCMs (in guinea-pig ileum). Conversely, even though BCM-7 still has the highest activity among other bovine BCMs in mouse vas deferens, human BCMs showed higher activity than all types of bovine BCMs that were discussed in the review [36]. µ-opioid receptor ligands or agonists, including morphine, have the ability to be used in the treatment of pain or analgesics. Therefore, much progress has been made in this field to produce analgesic effects through the central nervous system in the presence of µ-opioid receptor agonists. With their ability to bind to µ-opioid receptors in the central nervous system, BCMs have been shownt to exhibit sedative activity in addition to analgesic effects. BCMs of the BCM-4, BCM-5, and BCM-6 types show this analgesic effect for a period of 45 min, while BCM-7 has been demonstrated to show the same effect for 90 min or longer [37,38]. These food-derived opioid peptides can alter the opioid receptor pathway and contribute to various diseases and abnormalities. BCM-7, like the other β-casomorphines, an acknowledged µ-opioid receptor agonist, possesses several physiological properties [39]. Compared to BCM-7, the BCM-9 peptide exhibits opioid properties, but its binding affinity for μ-opioid receptors is approximately one-fourth that of BCM-7 [40]. Additionally, due to its status as a µ-opioid receptor agonist and the presence of these receptors along the gastrointestinal tract (as well as away from it), BCM-7 can penetrate the gastrointestinal wall and get into the blood circulation [41,42]. 

It is thought that BCM-7 is responsible for potential adverse health outcomes in humans, driven by its physiological impact. These include an increased risk of metabolic disorders such as diabetes mellitus, cardiovascular issues, neurological diseases (such as autism, and schizophrenia), immune events. There is also a possibility that elevated β-casomorphin-7 levels in the urinary tract system are linked to sudden infant death syndrome [8]. Table 1 presents studies elucidating the relationship between these disorders and BCM-7, categorizing the types of studies conducted. In addition, this review article aims to explore the potential of BCM-7 to manipulate disease mechanisms. In this context, data from literature have been compiled.

BCM-7 is currently highlighted in discussion of human health because it can be generated with A1 milk digestion, which is globally consumed. It is associated with very distinct health diseases, making BCM-7 a common topic of discussion. This review tries to evaluate how deeply these diseases are related to BCM-7. Specifically, considering the fact that some of these diseases are not closely correlated, this is an important topic research. However, it has not been studied with certainty. This review aims to provide some recent information on these diseases and how strongly BCM-7 is associated with in terms of its potential mechanism and biological character.

## 2. β-Casomorphin-7’s Direct Interaction with Certain Pathways

### 2.1. GLUT2 & GLUT4 Relation 

BCM-7 can have immune-altering effects and participate in immune-related events. Nonetheless, β-casomorphin (BCM-7) might be a trigger for type 1 diabetes through certain pathways. In this disorder, β-cells play a crucial role in autoantigens related to specific glucose transport (GLUT2), and the five amino acid sequences are identical to those found in bovine milk. This similarity is often described as a molecular mimicry [43,44]. To investigate this, one person from each family was tested to see if the individual had diabetes at one point in time. Subsequent tests confirmed that T lymphocytes exhibited cross-reactivity against β-casomorphin [45]. Additionally, a hypothesis was presented that the epitope structure on GLUT2 of the β-cells of the pancreas, which interferes with glucose transport at high Km value, is molecular mimicry by BCM-7 [44]. According to this hypothesis, BCM-7 stimulates the production of autoantibodies by interacting with GLUT2 epitopes. These autoantibodies, in turn, inhibit GLUT2 activity, preventing glucose transportation into the cell. Consequently, the BCM-7 is a potential trigger for diabetes mellitus [43]. BCM-7 can disrupt insulin hormone transmission regulation and impact metabolic processes related to insulin’s action. BCM-7 can influence the overall glucose distribution in the body through its opioid-like mechanisms. The μ-opioid receptor agonist character activates the insulin objective cell membrane receptors. Activation of these receptors may impede the phosphoinositide 3-kinase signaling pathway (PI3K). The PI3K pathway plays a role in converting the GLUT4 (glucose transporter 4) into its active form, facilitating glucose transport into the cell and thereby increasing glucose concentration in the cell. Conversely, inhibiting the PI3K pathway negatively affects the translocation of glucose into the cell, leading to elevated blood glucose levels. Due to that event, diabetes mellitus is triggered [44,46]. Thus the GLUT2 protein is found in sites such as the liver, pancreas, and small intestine, while the GLUT4 protein is found in adipose tissue, skeletal muscles, and the heart [47]. Given the potential interaction of BCM-7 with GLUT2 and GLUT4, this peptide can potentially show activity in different tissues or locations in the body.

### 2.2. TH-2 (CD+4) Pathway 

An earlier study investigated the inflammatory-inducing effect of BCM-7 in an animal model and indicated that both BCM-5 and BCM-7 induced inflammatory responses in the intestine of mice, potentially through the TH-2 (CD+4) pathway [48]. BCM-7 and BCM-5 supplementation orally enhanced certain inflammatory cytokines, such as IL-4 and histamine, and boosted the immune response in mice.

Researchers detailed that, a A2/A2 feeding group exhibited immunomodulatory effects by altering the levels of specific proteins (such as CD4/CD19) in B and T cells. The results suggested that diets focused on A2 milk could mitigate the negative effects of age-related immune changes such as susceptibility to infections and vaccination response [49]. The presence of BCM-7 in intestinal regions might trigger an undesired immune response through signals from T helper cells. The specifics of this BCM-7-related interaction are still unclear, but it is advisable to consider this potential interaction when investigating inflammation caused by casein peptides.

### 2.3. Relationship with GSH 

BCM-7 can also trigger a state of oxidative stress within the system. In situations where this stress is induced, the level of glutathione (GSH), which functions as an antioxidant in the human body, becomes depleted. The synthesis of GSH in the body occurs based on the presence of the amino acid cysteine in the environment [44]. This amino acid participates in the composition of the glutathione molecule and safeguards tissues from damage by binding to reactive oxygen species that cause oxidative stress [44]. 

Research on nervous system diseases (such as autism and schizophrenia), which develop due to low GSH levels in the brain, indicates that BCM-7 binds to the excitatory amino acid transporter 3 (EAAT3) carrier. EAAT3 is responsible for transporting cysteine amino acids into the cell. However, due to its opioid effect, BCM-7 prevents the uptake of this amino acid into the cell, hindering the production of GSH, which is essential for protecting the cell from stress. In a study with a double-blind randomized process involving forty-five individuals, it was demonstrated that consumption of milk containing A1-like variants significantly affects GSH levels. This decrease or disturbance of blood GSH level could potentially lead to the development of neurodegenerative diseases [50].

Additionally, researchers showed that a decrease in GSH concentration due to BCM-7 binding induced non-apoptotic, iron-dependent ferroptosis in the β-cells of the pancreas [28]. To counteract this ferroptosis effect, administering an opioid agonist molecule could be considered, as this approach has been shown to have a positive impact on GSH concentration. 

## 3. Clinical Studies and Potential Diseases Associated with BCM-7 

Studies investigating diseases linked to BCM-7 and A1 milk are given in Table 1.

**Table 1:** Studies investigating diseases linked to BCM-7 and A1 milk.

BCM-7 Related Diseases	Study Type	General Results and Outcome	References
Lactose intolerance	In vivo	The A1 milk consumer group generally had higher maldigestion compared to the A2 milk group.	[51]
Bowel-related disease	Randomized controlled trial	Among 387 toddlers, one A2-only milk, and one traditional milk group were created to investigate the gastrointestinal benefits of A2. The A2 milk group’s parents reported less intestinal constipation and increased GI comfort.	[52]
Central nervous system-related diseases	Animal study	The effect of consumption of A1 and A2 caseins on rat behavior was observed with A1/A2 diet groups. Rats fed with A1 casein showed high depressive-related behaviors and disturbance in neurochemical status.	[53]
Digestive discomfort	Randomized double-blind trial	In a small group of individuals (25-45) with lactose intolerance, the A2 milk group showed a lower number of symptoms compared to the traditional milk group.	[54]
Digestive discomfort	Randomized double-blind crossover trial	33 randomly assigned subjects that are 19-50 years old were fed with only A2 β-casein, conventional, lactose-free, and jersey milk. Compared to the group consuming other types of milk, the group consuming A2 had significantly fewer symptoms of abdominal pain, bloating, and diarrhea.	[55]
Inflammation/allergy	Animal study	Specific pathogen-free male BALB/c mice were fed a balanced diet and A1/A1 and A2/A2 β-casein milk for 30 weeks. According to the study, 30 weeks of feeding A1/A1 β-casein milk caused Th2-induced allergic airway inflammation. A2/A2 β-casein milk did not cause inflammation in this airway, but rather played a protective role for allergy and asthma.	[56]
Digestive disorders	Animal study	Aged Balb-c mice were fed with A1A2, A2A2, and standard laboratory pellet diet. The study did not show very specific results, but mice fed A1A2 milk showed an increase in CD3+ T cells involved in epithelial barrier maintenance compared to mice fed A2A2. Especially when the gastrointestinal tract is exposed to A2 β-casein, the aging mouse model has been shown to positively affect intestinal immunology and morphology.	[57]
Lactose intolerance	Randomized double-blind crossover trial	A 5-day study in lactose-intolerant preschool children compared the consumption of traditional (A1A2) milk with milk containing only A2 β-casein. Gastrointestinal symptoms and problems with stool frequency and consistency were reduced and normalized in child subjects fed milk containing only A2 β-casein. Furthermore, pediatric subjects consuming milk containing A2 β-casein had significant increases in serum levels of interleukin-4, immunoglobulins G, E, G1 and glutathione compared to subjects consuming conventional milk.	[58]
Diabetes melitius	Animal study	An animal study was performed to determine the A1 milk consumption (BCM-7 derived) effect on diabetes over generations of rats (starts from F0, ends at F3 generation). At the last F3 generation (week 30), the number of diabetic rates were double in the A1 group compared to the A2 gorup.	[59]
Central nervous system-related diseases	Double-blind randomized cross-study	Levels of GSH according to consumption of A1/A2 milk were investigated from the perspective of association between lower GSH levels and neurodegenerative diseases. It was found that the A1 consumed group showed lower blood GSH levels, potentially suggesting that BCM-7-based GSH disturbance can lead to neurodegenerative diseases and cell differentiation diseases	[50]
Digestive discomfort	Double-blind, randomized crossover study	A Small group of individuals (41 female) divided into two gorups: the A1 and A2 diet groups. The A1 group had abdominal pain and stool consistency, while the A2 diet group did not show such a correlation.	[29]
Bowel-related disease	Animal study	Consumption of A1/A2 milk on mice gut inflammatory response was investigated. A1 milk induced inflammatory cytokines and certain antibodies compared to the A2 diet group.	[60]
Bowel-related disease	Animal study	Consumption of different casein types (A2 A1) and how it alters the gastrointestinal transit were investigated. The A1 consumed group showed a significant delay when compared to the A2 diet group. The opioid-like activity of A1 was a priority in the research.	[31]
Central nervous system-related diseases	Human study	In a study with 90 infants, 37 were breastfed, and 53 were fed cow milk formula. The research indicated that a problem in the elimination of bovine β-casomorphin-7 is a risk factor that hinders the hypothesis of psychomotor development.	[41]
Cardiovascular diseases	Animal study	60 rabbits were fed A1 and A2 caseins for 6 weeks. The samples were collected at weeks 0, 3, and 6. A1 casein produced higher serum cholesterol and the thickness of the aortic lesion was higher in A1-fed rabbits. As a result, A1 casein is an atherogenic compound.	[61]
Diabetes melitius	Comparative study	The study examined the incidence of diabetes in children aged <14 years ol from 10 countries who consumed A1 variant cow milk protein. As a result of the study, especially in Iceland consumption of A1 variant cow milk protein is related to the occurrence of diabetes mellitus.	[62]
Milk allergy	Human study	26 children aged 5-9 years old were fed nutritive casein formula, and their skin reactions to cow’ s milk as a result.	[63]
Milk allergy	Human and in vitro study	14 healthy individuals with no history of cow’s milk allergies were tested. An allergic reaction to histamine release in the presence of BCM-7 has been demonstrated with a reaction on the skin after 15 min.	[64]

### 3.1. Lactose Intolerance

Lactose, a carbohydrate present in milk, leads to lactose intolerance, a digestive disorder characterized by the formation of undesirable symptoms when the body is unable to digest it [65]. Digestive system disorders, which are directly related to milk consumption, are not solely dependent on lactose status; they also depend on the type of caseins present in the milk, whether A1 or A2 [44]. In individuals with lactose intolerance, the transmission time of milk after digestion in the stomach is directly proportional to gastrointestinal symptoms [66]. Based on these associations, a randomized crossover trial was conducted involving the consumption of A1/A2 milk among lactose-intolerant individuals [51].

Researchers have indicated that A2 milk is generally easier to digest than A1 milk, and that individuals with lactose intolerance may not experience any gastrointestinal digestive problems when consuming A2 milk [55]. A study [30], involved 45 individuals with lactose intolerance issues who consumed only A2 milk and A1-A2 milk with an unknown, random 2 × 2 crossover method, with each treatment period spanning 14 days. The results revealed that lactose-intolerant individuals who consumed only A2 milk experienced fewer digestive disorders compared to those who consumed A1/A2 milk. In another trial, 10 individuals were divided into two groups to consume 75% A1 / 25% A2, and 100% A2 milk. The MRI analyses were applied to identify the milk quantity in the stomach at 0, 10, 30, 60, and 120 min after consumption. The transmission difference indicates that the first group (75% A1 milk consumers) had a lower amount of milk in their stomachs, compared to pure A2 consumers. Furthermore, the A1/A2 milk consumers reported a higher amount of symptoms (20) compared to A2 milk consumers (12). The research suggests that the difference could be a contributing factor to the higher occurrence of gastrointestinal symptoms in lactose-intolerant individuals after A1 milk consumption. Overall, lower rates of gas production and fewer digestive system disorders were observed in lactose-intolerant individuals consuming A2 milk [51].

### 3.2. Milk Allergy

Cow milk allergy (CMA) is a complicated condition that has sometimes been misdiagnosed, often being confused with other conditions like lactose intolerance. However, these two diseases differ in their mechanism, and distinct treatment methods are required [67]. CMA is typically divided into two different groups: IgE-mediated allergy and non-IgE-mediated allergy. The immunopathological mechanisms underlying non-IgE mediational allergy are not well understood, thus hampering the development of reliable diagnoses [67].

In contrast to non-IgE mediational allergy, which has a delayed onset, IgE mediational allergy triggers the release of histamine and other mediators, leading to a rapid onset of symptoms [68]. Symptoms of IgE-mediated CMA symptoms include eczema and urticaria. Caseins are among the main allergens responsible for CMA, and this condition most commonly occurs in early childhood [69]. β-casomorphins have the potential to induce allergic reactions by triggering histamine release from immune cells [44]. Researchers demonstrated that [63] BCM-7 affects histamine release in humans. In their study, pseudoallergic blistering and glowing skin reactions were observed when BCM-7 was intradermally injected into healthy volunteers. Additionally, BCM-7 induced the release of histamine from peritoneal mast cells (PMC) of rats and isolated human leukocytes [64]. In another study conducted by the same group, it was suggested that individuals hypersensitive to histamine-releasing peptides may play a role in the intolerance of casein formulas [63]. A researcher found that [70] incubating peripheral blood mononuclear cells (PBMC) with peptide extracts from A1 cow milk (BCM-7) increased the gene expression of the μ-opioid receptor (MOR) while decreasing DPP4 (dipeptidyl peptidase 4) enzyme’s gene expression in children aged <14 years old with atopic dermatitis.

### 3.3. Diabetes Mellitus (Type-1)

Type-1 diabetes (DM-1), an autoimmune disorder, is characterized by the loss of functionality of the β-cells in the pancreas, which produce insulin [71]. This condition is marked by autoimmune destruction of these cells by the T cells or macrophages [72].

DM-1, formerly referred to as diabetes mellitus with insulin-dependence, is the second most common disease among children, particularly those under 14 years old [43,44]. Besides genetic predisposition, other environmental and nutritional factors, especially cow milk, along with the autoimmune systems, create a significant change in the pathogenesis of individuals experiencing insulin deficiency due to this disease. An individual’s diet is a crucial factor in the development of this condition. For instance, daily cow milk consumption by infants and older individuals has been correlated with the incidence of DM-1 in genetically at-risk individuals [43]. In a prior study, researchers demonstrated [73] an enhancement in the levels of antibodies against β-casein protein in diabetes mellitus patients. Consequently, they hypothesized that β-casein might contribute to the development of diabetes mellitus. This hypothesis gained support through an animal model study, showing that the administration of A1 protein (BCM-7) to non-obese diabetic mice promoted the development of diabetes, while A2 protein (BCM-9) did not induce such a situation [43]. 

Furthermore, in another study [62], it was reported that the incidence of diabetes in children < 14 years of age across 10 countries who consumed milk protein was reported. The study showed a lower incidence of type 1 diabetes and heart disease among Icelandic children who primarily consumed A2 protein. BCM-7 peptide was evaluated as a risk factor for this disease, and subsequent studies conducted after this investigation supported this notion [74,75].

Additionally, two separate research studies comparing cow milk consumption and disease incidence in 16 and 19 different countries among children aged <14–15 years obtained similar results [74,75].

Normally, differences in diabetes mellitus incidence between countries are attributed to extrinsic factors like cow milk consumption. However, the children in all these studies either possess or were exposed to the same extrinsic factor. Therefore, variations in milk protein consumption suggest a connection between these diseases.

### 3.4. Cardiovascular Diseases

Ischemic heart disease (IHD) is a major cardiovascular condition resulting from the narrowing of the coronary arteries that supply blood to the heart muscle. This constriction in the coronary arteries leads to inadequate blood and oxygen supply to the heart. Additionally, A1 β-casein has a strong association with IHD [75]. BCM-7 has been linked to the oxidation of human LDL (low-density lipoprotein), a risk factor for heart disease. Oxidized LDL adheres to arteries, leading to plaque formation [76].

Researchers showed that [77] BCM-7 supports LDL oxidation in individuals at risk of heart disease. Oxidized LDL, induced by oxidative stress, plays a critical role in atherosclerosis development in humans [78]. In another studying involving men aged 30 to 69 years ol from 16 different countries, found an association between the consumption of A1 milk (excluding cheese) and the risk of heart disease [75]. Analyses showed that BCM-7 was a significant trigger for cardiovascular disease in developed countries. It was observed that the death rate increased over time in individuals consuming β-casein A1. Additionally, numerous animal experiments involving rabbits, pigs, monkeys, and rodents have discussed the relationship between β-casein consumption and hypercholesterolemia or atherosclerosis [44]. For instance, rabbits fed A1 milk exhibited higher cholesterol levels and a more extensively covered aortic surface compared to those fed β-casein A2 milk [61].

### 3.5. Potential Effect on Central Nervous System

In the central nervous system (CNS), opioids can permeate the blood-brain barrier (BBB), leading to the activation of specific receptors and subsequent alterations in pathways in nearby regions. BCM-7 is indeed classified as an opioid peptide, although there is currently no concrete research and evidence regarding the direct impact of BCM-7 on the nervous system. Nevertheless, some indirect effects can be observed or hypothesized. Glutathione (GSH) plays a pivotal role in the nervous system by acting as an antioxidant that maintains oxidative balance within the region. As individuals age, the level of GSH tends to decrease, increasing the risk of neurodegenerative disorders [79]. Based on this information, a previously mentioned study discussed the effect of the imbalance of GSH levels on neurodegenerative diseases [50]. Blood tests indicated that individuals consuming A1/A2 milk had higher levels of BCM-7 and lower blood GSH levels, compared with exclusive A2 milk consumers. These findings suggest that this opioid peptide may disrupt the redox state in the individuals and potentially impact the neuronal stem cell and neurogenesis, as redox balance is crucial for the differentiation of neuronal cells [80]. A study examined several infants who were either breastfed or formula-fed with bovine milk-containing formulas [41]. The comparison between artificial and naturally fed infants showed that most infants that were artificially fed showed psychomotor development delays and increased immunoreactivity of blood plasma BCM-7 after feeding. BCM-7 levels in infants can be vary due to differences in metabolic rates, which are higher in most infants. If the metabolism rate is slow, there is a higher chance of developing delayed psychomotor development due to the higher levels of blood BCM-7 between the feeding sessions as observed in previous research [48]. 

Higher levels of BCM-7 in the blood plasma and urine samples were correlated with psychomotor delays and potentially the future development of specific conditions like autism [41,81,82]. However, the correlation between BCM-7 and autism requires further understanding and in-depth research. An animal experiment was performed to further investigate the A1 casein’s effect on behavior in rats [53]. Three groups were formed: casein-free group, casein-rich group, and control group (receiving commercial milk). Rats in the casein-rich milk group, a milk group exhibited depressive-related behaviors and neurochemical changes, whereas the high A2 group or the casein-free group did not display similar symptoms during the study. Opioid receptors in the brain region were also upregulated, a phenomenon not observed in the A2 and non-casein groups. This might be an alternative perspective to approach the current discussion on the activity of BCM-7 against the central nervous system. All these results can indicate the BCM-7’s influence on the CNS via its high affinity for opioid receptors found in both the gastrointestinal region and the brain. It also needs to be emphasized that a majority of these studies are based on in vivo rat studies, which is another indicator of the deficiency of existing studies. Even though there is not enough clear evidence to show BCM7’s direct effect on the CNS, the abundance of current studies shows that BCM-7 should be investigated further to confirm and expand these negative effects influenced to the brain.

### 3.6. Gastrointestinal Issues and Intestine-Related Diseases

The digestion of BCM-7 can give rise to gastrointestinal (GI) issues. These undesirable factors primarily include lactose intolerance, abdominal swelling and pain, alterations in stool consistency, a reduction in intestinal bacterial mass, an increase in mucin production, and an inflammatory response (TH2 response), some of which have already been discussed [83].

In preclinical animal model studies comparing A1 and A2 variants, it was observed that the A1 variant might lead to a higher rate of inflammation. In the study, mice were fed with A1 casein-like cow’s milk to visualize the inflammatory response [48]. Under the study’s conditions, four animal groups were created and fed milk containing β-casein genotypes A1A1, A1A2, and A2A2 and compared to the control. The gut immune response indicated that consumption of A1 variants probably increases myeloperoxidase enzyme, total interleukin-4, and immunoglobulin G, G1.

When compared to mice fed A2, increased intestinal permeability and production of interleukin-4 (IL-4) by T helper type 2 cells [84], which are referred to as inflammatory markers, were observed. This implies that BCM-7 triggers inflammation in the intestines. 

In addition, the activity of the myeloperoxidase enzyme in the azurophilic lysosomal granules of neutrophils is associated with the number of neutrophils in the histology section of the gastrointestinal tract, making this enzyme act as a biomarker in the intestines [85].

An increase in MPO activity was observed after the ingestion of bovine milk containing A1 casein. In an animal model study designed to illustrate differences in nutrition of different A1 and A2 caseins, the gastrointestinal passage time was longer in the A1-consuming group [31].

As a result, it is hypothesized that the alteration in gastrointestinal passage time due to the BCM-7 amino acid chain may affect the balance of the gut microbiota [31,86]. Apart from this, studies have shown an effect on the gastrointestinal tract, reduced stool mass, bacterial mass, and gastrointestinal motility [87]. The other BCM-7 actions are decreasing the gastrointestinal motility and resembling the morphine activity. These effects were demonstrated in a 2003 human-based study. In the study, newborn infants were fed cow’s milk-based formula or their normal mothers’ milk. The results showed abnormal anatomical structures known as anal fissure, and constipation formed in the formula-fed group, while the human milk group experienced no such constipation or anal fissure forming [88]. Therefore, these results suggest the potential influence of BCM-7 on its opioid characteristics following consumption.

The difference in BCM-7’s amino acid chain can affect the bacterial population in the microbiome [89]. In a study, it was shown that BCM-7 directly activates mucin synthesis in goblet cells due to its opioid character. Excessive mucin production can, in some cases, lead to the deterioration of the protective barrier structure and the interaction of the invaders with the microbiome. This thick layer of mucus, often referred to as slimy fluid, is primarily composed of mucin proteins produced by goblet cells, and it serves as a protective barrier against potential infections in the intestinal epithelial cell membranes [32].

The mucus layer plays a crucial role in maintaining intestinal health by acting as a barrier. However, an increase in the production of this layer in individuals with leaky gut (permeable bowel syndrome) syndrome can exacerbate the progression of the disease. A study demonstrated that BCM-7 increased mucin production by binding to endogenous opioid receptors [60]. Therefore, the consumption of A1 casein poses a significant risk for individuals with permeable bowel syndrome [44,89].

In another study conducted on DHE rat intestinal cancer cell lines in rats, it was reported that BCM-7 affected mucin secretion by enhancing the rMuc2 and rMuc3 mucin gene expression [32,60]. Well-known factors are glucose and calcium ions affecting opioid peptide epithelial transport, and BCM-7 has been analyzed in the application of glucose or calcium ions by the Caco-2 system.

The uptake of BCM-7 from the intestine was investigated in an in vitro experiment on Caco-2 cell medium. In this medium, which contained Caco-2 cells, a high concentration of glucose and calcium was added, and the rate of BCM-7 uptake was observed. In the experiment, the presence of diprotin A, along with high concentrations of both calcium and glucose, led to an increased uptake of BCM-7. These results indicate that a high-sugar diet might increase the opioid intake from gut regions and cause further disturbances [90].

The effect of A2 β-casein in intestinal regions was studied in a global patent application and research [91].

It was observed that bowel movements and stool structure were better when compared to other ordinary milks. Most importantly, the amount of *Bifidobacterium* spp. in the human intestine increased with the A2 milk treatment. However, one limitation of this research is that the characteristics of the tested milk were unknown, so comparison of the casein types cannot be properly made. Still, the impact of A2 milk on the human intestine is observable. 

A recent study looked at the immunomodulatory effect of A2 β-casein in immunosuppressed BALB/c mice [92]. According to the result of this study, A2 β-casein can promote spleen lymphocyte proliferation and significantly increase macrophage phagocytic index and NK cell activity. Moreover, A2 β-casein can increase SCFA concentration and regulate the diversity and composition of intestinal microbiota and improve intestinal mucosal immune function in CTX-induced immunosuppressed BALB/c mice. This result further proves that A2 β-casein has the potential to act as a gut microbiota and immune modulator to reduce the harmful effects of CTX on the immune system. This study may be a pioneer in the literature for A2 β-casein to be included in the functional food field.

### 3.7. Sudden Infant Death Syndrome (SIDS)

The most common cause of death in infants is sudden infant death syndrome (SIDS). It occurs in infants between the end of the first month and the first year after birth [39]. After milk consumption, the enzymatic breakdown of casein forms smaller peptides. These peptides are rich in proline and they are highly resistant to proteolysis, so they can reach substantial levels in the stomach. After absorption from the GI tract, BCMs can easily pass the blood-brain barrier due to the infant’s immature CNS. Therefore, opioid peptides from digested milk may cause depression in the brainstem respiratory centers due to abnormal respiratory control and vagal nerve development in infants. According to a study, this has been shown to be contributing factor in apnea and sudden infant death, but also produces BCM-7 after the digestion of human milk. In this context, it is a situation that needs to be evaluated more and investigated at different scales [33,93]. Casein-derived elements have been found in the brain stem of human infants suffering from sudden infant death syndrome, and BCMs have therefore been generally proposed as a possible cause of SIDS [94]. However, whether the BCM-7 peptide is specifically responsible is not certain indication. In addition, higher blood levels of BCM-7 and lower activity of active serum dipeptidyl peptidase 4 (DPP4) were found in infants with an apnoea episode compared to healthy infants [95]. DPP4 is the only enzyme in humans that can break down BCM-7 into X-Pro dipeptide at the N-terminus position. In healthy children, the body increases DPP4 activity due to the high level of BCM-7. Indeed, it is undebatable that the consumption of human milk is more beneficial when compared to formula milk [96,97]. Still, more research needs to be performed to decrease the disadvantages of formula feeding, especially with the BCM-7 perspective. Studies in the literature have demonstrated injection of BCM-7 into the blood of young rabbits and rats might cause apnoea (irregular breathing) [98]. However, these results were only seen in adults rats fed high doses of BCM-7 adult rats that are fed BCM-7, while there are no definitive results associated with BCM-7 in neonatal rabbits. 

Additionally, in the Caco-2 cell monolayer, researchers have shown that peptides with a β-turn structure have higher permeability than those with a linear structure [99]. In a different study showed that the β-turn structure of BCM-7 may have a significantly higher transport coefficient. In this study compared to the combination of lactoferroxin A and BCM-7, and BCM-7 alone was shown to have high permeability through the Caco-2 intestinal monolayer [100]. Therefore, after digestion of bovine A1 milk by the mother, BCM-7 can pass into breast milk and cause life-threatening events in infants [41,101]. The same goal investigation should also be applied to human studies to identify the potential BCM-7 transmission from mother to infant, especially for mothers who are high consumers of bovine milk [41,102]. Further studies are required for this disease as there only limited number of studies that indicates the sudden infant deaths are due to the presence of BCM-7 or that can explain this situation.

## 4. Effect of A1 and A2 Caseins on Calcium Content and Potential Bone Health

The potential effects of BCM-7, as an opioid peptide, are currently being investigated to understand how they correlate with the overall effects of opioids in the body. As an example, certain µ-opioid receptor agonists, like morphine, alter the secretion of osteocalcin in osteoblastic cell lines [103]. When these cell lines are exposed to these molecules at abnormally high levels, the secretion rate of these osteocalcins seems to be reduced. Therefore, the accumulation of these opioid receptor agonists might decrease the osteocalcin levels and indicate an abnormality in bone formation, thus potentially leading to bone fragility. The specific interaction between bone abnormalities and opioids has not been investigated. For instance, a cohort study (61,433 women and 45,339 men) in 2014, in Sweden, was published on the interaction between fractures and milk consumption [104]. The cohort study points out that women who consumed more than two glasses of milk a day had a higher ratio of fractures, compared to women who consumed only one glass of milk. Analyze the results, the main reason behind the high rate of fractures might be the high amount of A1-derived BCM-7 levels, given that Sweden as the highest rate of A1 milk consumption [103]. Still, due to a lack of evidence and unclear reliability of the study, a clear comment cannot be made.

Even though not many studies have analyzed the calcium content in milk in comparison to casein type, some suggested that the distribution and content of calcium in milk can show diversity due to the different variations of β-casein and affect the milk-based products [105]. β-casein plays a major role in the casein micelle formation in milk, and they are known for their calcium-sensitive characteristics. Research indicates that the A2 version of BCM-7 has more ionic Ca+2 (unbound) as percentage of total calcium than the A1 version (6.9%, and 5.1% respectively). The difference between free calcium percentages might be due to the lower bound affinity of A2-derived casein micelle than A1-derived ones [106].

The free ionic calcium content in milk alters characteristics of both milk and dietary products. For instance, research aimed at analyzing the effects of A1 and A2 β-casein in the yogurt gel properties found that A2 milk created a lower strength of gel in yogurt production, potentially enhancing the digestion of the product [106]. In the aspect of the findings, it is expected that type of the casein alters not only the characteristics of milk but also the milk-based product. Especially when milk allergy and lactose intolerance cases are considered, it seems like a good strategy to focus on the β-caseins in the production of milk products. The role of calcium in both A1 and A2 milk should be investigated in more detail to confirm or decline these claims and take precautions if necessary. Future findings about the calcium content of the milk, especially from the perspective of A1/A2 variants, can be helpful the perspective of both change the industrial application of milk and the health outcomes.

## 5. Conclusions

From the review, it is clear that bovine milk is a vital source of energy and occupies an important place in children’s diets because of its nutrient-rich qualities, especially β-casein proteins. These proteins, vital for growth and health, include the common A1 variant, which results from a mutation at position 67 in the A2 variant. According to existing research, consumption of A1 casein cow’s milk poses risk attributed to the BCM-7 peptide formed during digestion, including type 1 diabetes, cardiovascular disorders, digestive disorders, allergen formation, and neurological disorders. Studies have attempted to show that BCM-7 peptide may cause changes in the molecular mechanisms of diseases. Although BCM-7 is generally studied with an emphasis on the opioid mechanism, there are also studies suggesting that it can cause conformational change and induce or reduce other protein mechanisms indirectly. However, no definitive and clear conclusions have been made about the BCM-7 peptide being the direct cause of these diseases, although studies have shown that it possesses the potential to do so. Moreover, the literature in this field is limited. More studies that provide a molecular understanding of BCM-7 and its effect on human health at the molecular level or clarity on its mechanisms should be conducted.

## 6. Future Perspective

BCM-7 stands out in the literature for its opioid-like properties and being an A1-type milk-derived peptide. However, research studies on this subject are still limited. We are aware that there are very few studies on A1 milk and BCM-7 peptide. Although there are several new and ongoing studies on the effect of BCM-7 on gastrointestinal sites, other important parts of human health involving the potential activity of BCM-7 are still poorly defined and absent in some studies. The number of studies based on diseases such as cardiovascular diseases, neurological disorders, and allergies, which have been at the forefront in recent years, is lacking. Therefore, more studies are needed in the future perspective. New studies will allow interpretations and inferences to be made on these topics and fill the gaps in the literature. Similarly, there are deficiencies in understanding of BCM-7 at the molecular level. While BCM-7 is typically studied with a focus on its opioid mechanism, there is limited literature demonstrating its potential to elicit conformational changes and indirectly trigger other protein mechanisms. The absence of studies elucidating these mechanisms at the molecular level is conspicuous in the scientific discourse. Investigating the signaling pathways associated with these mechanisms and elucidating the effect of BCM-7’s opioid properties on these pathways will serve to fill this gap in the literature. There have been recent studies comparing A2 milk or certain different diets with A1 milk. Naturally, the molecular mechanism behind these differences and results remains unexplained or under investigation due to lack of base data in the literature. Researchers in this field neet to focus future researchers efforts more on the molecular background and conduct clinical trials rather than comparison of current data. Large clinical trials should be conducted and sufficient data should be collected to conduct cell biology-based studies that will fill gaps in the study of BCM-7.

## Data Availability

Data availability is not valid for our study “BCM-7: Opioid-like Peptide with Potential Role in Disease Mechanisms”, this article does not generate new data and describes purely theoretical research. Since no data sets or analyses were performed during the current research, data sharing is not applicable for this article.
